# Bridging health and community: descriptive analysis of social prescribing for older adults in Cambodia

**DOI:** 10.1016/j.lanwpc.2025.101790

**Published:** 2026-01-15

**Authors:** Hitomi Kimura, Sovandara Kao, Sareth Khann, ChanPeou Phan, Daravuth Yel, Ada Moadsiri, Mikiko Kanda, Siwon Lee

**Affiliations:** a Healthy Ageing Unit, Division of Healthy Environments and Populations, World Health Organization Regional Office for the Western Pacific, Manila, Philippines; b Department of Psychology, Royal University of Phnom Penh, Cambodia; c World Health Organization Cambodia Country Office, Phnom Penh, Cambodia

**Keywords:** Social prescribing, Community empowerment, Older adults, Resource-limited settings

## Abstract

**Background:**

Social prescribing supports individual well-being and community engagement by linking people to local resources. In Cambodia, members of the existing Village Health Support Groups have been trained as link workers to deliver social prescribing activities by leveraging existing resources in a limited-resource setting. However, the nature and functioning of social prescribing in limited-resource settings remain poorly understood. This study aims to describe the implementation of social prescribing in Cambodia and examine how a social prescribing intervention for older adults was associated with improved access to healthcare, greater community support, and reduced loneliness, providing initial insights to inform future implementation research.

**Methods:**

A cross-sectional study was conducted across ten Cambodian provinces among 1200 older adults aged 60 and above between 1 December 2024 and 31 January 2025. We interviewed older adults in each of the following groups using a structured questionnaire: Group 1 (n = 400, received social prescribing), Group 2 (n = 400, did not receive but lived in areas with trained link workers), and Group 3 (n = 400, lived in areas without trained link workers). Descriptive analyses were conducted to summarise the demographic and contextual characteristics of participants across the three groups. Additional descriptive analyses were conducted for Group 1 to summarise the characteristics of social prescribing activities, and qualitative responses were thematically analysed. Logistic regression analyses were conducted to examine associations between social prescribing exposure and key outcomes. The primary outcomes were overall health status and loneliness. Secondary outcomes included consultation opportunities, healthcare access, unmet needs, and health status. Two comparisons were made: recipients versus non-recipients within trained areas (Group 1 vs Group 2) and non-recipients in trained versus non-trained areas (Group 2 vs Group 3). Models were adjusted for age, sex, marital status, education, household size, and IDPoor Equity Card status–a government measure of household poverty.

**Findings:**

Among those who received social prescribing, information was most commonly provided by Village Health Support Groups (82.7%) and village leaders (76.9%) and were most often delivered at home (55.2%). Referrals were mainly to health facilities (84.3%) and community activities (77.2%), and also included counselling at pagoda (20.6%), mental health support (21.4%), and daily life assistance (17.0%). 98.9% of participants reported that social prescribing was helpful. Enhanced health literacy, practical support, and improved psychosocial well-being emerged as themes from the qualitative analysis of the semi-structured interviews, health literacy, practical support, and improved psychosocial well-being emerged as themes from the qualitative analysis of the semi-structured interviews. Compared to Group 2, Group 1 was significantly associated with greater opportunities to consult with community supporters (multivariable-adjusted OR (aOR) 1.65, 95% CI 1.24–2.20, *p* = 0.00054), which remained statistically significant after Bonferroni correction. In contrast to Group 2, Group 1 was less likely to report poor healthcare availability (aOR = 0.73, 95% CI: 0.53–1.00, *p* = 0.048) and loneliness (aOR = 0.60, 95% CI: 0.37–0.97, *p* = 0.039), although these associations were not significant after Bonferroni correction.

**Interpretation:**

Social prescribing in Cambodia, delivered through existing Village Health Support Groups, appeared feasible and was perceived as highly helpful by older adults. Descriptive findings suggest that trained link workers may enhance community support in limited-resource settings. These insights offer an initial understanding of how social prescribing functions in low-resource contexts and can inform future implementation research.

**Funding:**

10.13039/100004423World Health Organization Regional Office for the Western Pacific.

**Research in context**

**Evidence before this study**

Evidence for social prescribing has expanded substantially, evolving from early findings in the UK on reduced primary care use to studies reporting improvements in mental health, management of long-term conditions, and economic outcomes. The evidence base now includes a wide range of interventions, including green or nature-based social prescribing. Although the number of randomised controlled trials is increasing, the evidence remains largely derived from high-income settings. As a result, the implementation and impact of social prescribing in low- and middle-income countries, including Cambodia, remain poorly understood.

**Added value of this study**

To our knowledge, this is the first study to describe the implementation of a social prescribing model for older adults in Cambodia. We conducted a cross-sectional study of 1200 older adults across ten provinces, comparing recipients of social prescribing, non-recipients in trained areas, and non-recipients in non-trained areas. We found that the intervention, delivered through existing Village Health Support Groups (VHSGs), was perceived as highly helpful by 98.9% of recipients. Recipients reported benefits such as health education (69.0%) and motivation for health check-ups (44.8%). Statistical analysis showed that recipients had significantly greater opportunities to consult with community supporters (adjusted OR 1.65, 95% CI 1.24–2.20, *p* = 0.00054), specifically VHSGs, volunteers, and village heads. While associations with reduced loneliness and improved healthcare availability were observed, they did not remain statistically significant after Bonferroni correction.

**Implications of all the available evidence**

Our findings suggest that integrating social prescribing into Cambodia's existing VHSG network is a feasible, contextually relevant, and low-cost strategy that aligns with the National Ageing Policy. By leveraging trusted community structures, this model effectively connects older adults to local assets and promotes community engagement. Although the associations with reduced loneliness and better healthcare access require confirmation through future longitudinal and cost-effectiveness studies, the results indicate that trained link workers can enhance community support. This study offers a scalable template for other low- and middle-income countries confronting the dual challenges of rapid population ageing and limited resources.

## Introduction

Cambodia is a lower-middle-income country experiencing population ageing, urbanisation, and rapid economic development.^1^ These changes contribute to an increased burden of noncommunicable diseases (NCDs).^2^ Cambodia has introduced several national strategies addressing NCDs, ageing, mental health, and primary health care to respond to these emerging challenges.^3^, ^4^, ^5^, ^6^

In 2019, people aged 60 years and older accounted for 8.86% of Cambodia's population, an increase of about 60% compared with the 2008 Census, and this proportion is projected to rise to 23.17% by 2050.^7^ Life expectancy also increased markedly between 2008 and 2019, from 60.8 to 74.3 years for men and from 63.7 to 76.8 years for women.^7^

Cambodia does not yet have a comprehensive national long-term care system, and older adults largely rely on informal care from family and community networks.^4^ Health services are overseen by the Ministry of Health, while social welfare and older people's associations fall under the Ministry of Social Affairs, Veterans, and Youth Rehabilitation, with variable levels of coverage and activity across the country.^4^^,^^8^ Civil society organisations provide complementary support in selected areas. Further research is needed to effectively transition Cambodia into a healthy ageing society.

The National Ageing Policy outlines nine priority areas: financial security; health and well-being; living arrangements; enabling environments; active ageing and older people's associations; intergenerational relations; prevention of elder abuse, neglect, and violence; emergency situations; and preparing younger populations for ageing.^4^ Achieving these priorities requires coordinated, multisectoral action. The policy is guided by two overarching goals: enabling older people to participate in community life with dignity and autonomy, and preparing younger generations for healthy ageing.

Social prescribing is a means for health-care workers in primary care settings to connect patients to a range of non-clinical community services to improve their well-being.^9^ It takes a holistic approach, addressing social determinants of health rather than symptoms alone. A global Delphi study defines social prescribing as a process through which trusted individuals in clinical and community settings identify non-medical, health-related social needs and connect people to community-based, non-clinical supports through a co-produced social prescription to improve health, well-being, and social connection.^10^

This broader, community-oriented approach could assist in advancing Cambodia's National Ageing Policy by strengthening older people's links to community assets, enabling them to engage in family, community, economic, social, religious and political life with dignity.^4^^,^^11^, ^12^, ^13^, ^14^ Social prescribing could also involve younger volunteers and community members, supporting intergenerational exchange and helping prepare future generations for healthy ageing.^4^^,^^11^, ^12^, ^13^, ^14^

Social prescribing was introduced in Cambodia in 2022 in response to heightened mental health needs during the COVID-19 pandemic. Mental health specialists from the Royal University of Phnom Penh, supported by the World Health Organization, trained Village Health Support Groups (VHSGs) to be “link workers”. The VHSGs comprise a range of individuals, including village chiefs, who are local leaders responsible for village administration and communication with higher government levels; vice chiefs, who assist the chief; other village members, who may serve secretarial or statistical roles; and women's affairs representatives, who focus on community development and issues affecting women and children. The link worker training curriculum combined practical counselling skills and screening tools with modules on stress, anxiety and depression management, dementia care, and mindfulness techniques such as meditation, breathing exercises and progressive muscle relaxation.

VHSGs identified older people with unmet social or health needs using a programme-specific questionnaire, provided information, referred or accompanied participants to health and social services, and conducted home follow-up. Through collaboration with village authorities, commune councils, pagodas, and district social welfare offices, they mobilised local resources, supported social welfare certification, and escalated complex cases. This community-driven model complemented existing health-sector referral pathways and was well suited to addressing social determinants of health.

To date, no studies have documented how social prescribing is implemented in Cambodia, and there is no standardised approach to evaluating its outcomes in this context. This study therefore aimed to describe the implementation of a novel, low-cost social prescribing model for older adults delivered through existing VHSGs acting as link workers, and to explore its associations with health and community-related outcomes. We hypothesised that older adults who received social prescribing would report greater opportunities for consultation, improved access to healthcare, and reduced loneliness compared with those who did not. As a supplementary analysis, we examined whether the presence of trained link workers was associated with these outcomes at the community level.

Given the global evidence of social prescribing's benefits, particularly in fostering stronger community connections and improving the well-being of older adults, Cambodia's model offers valuable initial insights into implementing this innovative approach in low-resource settings.^12^^,^^13^^,^^15^

## Methods

### Study design and setting

This cross-sectional observational study was conducted across ten Cambodian provinces where link-worker training for VHSGs had been implemented in 2022 as part of a national initiative to strengthen community-based support for older adults. The training districts had been purposively selected in coordination with provincial governors to ensure implementation readiness, facilitate programme monitoring, and maintain consistent operational procedures across provinces. The training was designed based on WHO's toolkit on how to implement social prescribing, which had been translated into Khmer and locally adapted for use in the Cambodian context. Data for the present study were collected between 1 December 2024 and 31 January 2025 and included community-dwelling adults aged 60 years and above.

### Sampling strategy and participant groups

Across the ten provinces, each of which had implemented link-worker training in 2022 and 2023, we included all trained districts for sampling and randomly selected one additional district without training from each province (Supplementary Fig. S1 and Supplementary Table S1). From every trained and non-trained district, four communes were randomly selected. Participants were older adults aged 60 years and above, or their family members when older adults were unavailable. In communes within trained districts, participants were sampled in two categories: those who had received social prescribing support from trained link workers (Group 1) and those who had not (Group 2). In communes within non-trained districts, only participants who had not received social prescribing were included (Group 3). Ten participants were randomly selected from village records maintained by Village Health Support Groups (VHSGs) for each relevant category in every commune, yielding a total sample of 1200 participants evenly distributed across three analytic groups: 400 recipients of social prescribing in trained districts (Group 1), 400 non-recipients in the same trained districts (Group 2), and 400 non-recipients in districts without link-worker training (Group 3). Temporary visitors and individuals supported by non-trained link workers were excluded. Before data collection, the research team established rapport with potential participants and provided a clear explanation of the study objectives, procedures, and confidentiality to ensure informed and voluntary participation. No eligible participant declined participation.

This sample size was selected to ensure adequate statistical power; assuming a baseline prevalence of loneliness of 30–40%, a two-sided alpha of 0.05, and 80% power, the study had sufficient power to detect small-to-moderate differences between groups a two-sided alpha of 0.05, and 80% power, the study had sufficient power to detect small-to-moderate differences between groups.

### Exposure ascertainment and data collection

Exposure to social prescribing was determined using VHSG administrative records. Eligibility was identified from rosters documenting residents' needs across physical, mental, financial, and social-welfare domains, and receipt of social prescribing was verified through programme logs documenting the delivery of social prescriptions. Data on potential confounders and outcomes were collected using a structured, paper-based questionnaire administered face-to-face in the local language by trained interviewers at participants’ homes or other community settings. The questionnaire captured demographic characteristics, household conditions, consultation opportunities, healthcare availability, unmet needs for health and social care, self-rated health, loneliness, and where applicable, the content and delivery channels of social prescribing (Supplementary Table S2). The instrument has not been formally validated in Cambodia; however, items were aligned with widely used international measures where feasible and adapted to the local context to accommodate older adults with limited literacy. The Khmer questionnaire was pre-tested for clarity. Semi-structured qualitative questions were also included to gather perceptions of the programme, perceived benefits, and suggestions for improvement.

### Variables and statistical analysis

#### Participant characteristics

First, we described the demographic and contextual characteristics of older adults across three groups based on their exposure to social prescribing and trained link workers. All variables were collected using a structured, paper-based questionnaire administered face-to-face in the local language by trained interviewers (Supplementary Table S2). For continuous variables such as age and the number of household members, we calculated the mean and standard deviation (SD) for each group. We summarised categorical variables as counts with corresponding percentages. The categorical variables were sex (female, male, or others), marital status (single, married, widowed, or divorced), and household composition (alone, with spouse, children, or other relatives), education level (none, pagoda, primary school, secondary school, or higher education), IDPoor Equity Card holder (yes, or no), monthly household income (less than 100 USD [United States Dollars]), 100–<300 USD, 300–<500 USD, 500–<700 USD, 700–<900 USD, or more than 900 USD), sewage system at home (clean water only, sewage system only, both, neither), access to healthy food and functional cooking device (yes, or no), enough space in the house to live comfortably (yes, or no), home isolated from external stressors (ex. noise, pollution, hostile weather, storm, flood dry and heat waves) (yes, or no), and safe at home and neighbourhood (yes, or no) were summarised as frequencies and percentages. Poverty status was assessed using the Government of the Kingdom of Cambodia's Identification of Poor Households Programme (IDPoor). This system serves as the official national registry for poverty targeting.^16^ IDPoor Equity Card, which makes them eligible for state-provided support, including fee exemptions at public health facilities through the Health Equity Fund.^16^

### Description of social prescribing content and delivery

Next, we described the content and delivery channels of social prescribing among participants who received social prescribing, with the aim of providing a clearer understanding of the intervention's structure, operational processes, and on-the-ground implementation of social prescribing in low-resource settings. All variables were collected using a structured, paper-based questionnaire administered face-to-face in the local language by trained interviewers (Supplementary Table S2). The overall recall rate was calculated as the proportion of participants who reported remembering having received social prescribing, presented as a percentage. Among those participants, we examined the following factors, and calculated the frequency and percentage. These factors included the sources of information (commune health centres, commune women's councils, village members, village health support group, family or direct consultation by older person, religious group, village head, volunteer, or neighbour), first impression of social prescribing (positive, neutral, or negative), topic for consultation (physical problem, mental problem, pain, mobility and falls, cognitive decline, medication management, financial insecurity, elder abuse, isolation and loneliness, access to health care, or nutrition), location conducted social prescribing (at home, pagoda, health centre, commune hall, or village office), referred service (health facility, social group and community engagement, educational classes, counselling at pagoda, mental health support, peer support, financial advice, transportation, or daily life support), frequency of follow up (every month, every 3 month, every 6 month, or seldom or never), and overall helpfulness of social prescribing (yes, or no).

### Qualitative analysis

For the qualitative component, semi-structured interviews were conducted to explore participants' perceptions of social prescribing, including its perceived benefits, helpfulness, and areas for improvement. This qualitative approach was intended to provide contextual data to help interpret and expand upon the quantitative findings. NVivo 15.2.0(21) was used to assist with qualitative data management and thematic coding. The interview transcripts were thematically categorised to identify recurring themes related to the programme's perceived benefits, helpfulness, and recommendations.

### Analyses of associations between social prescribing exposure at individual (Group 1 vs 2) and community (Group 2 vs 3) levels and key outcomes

Lastly, we conducted logistic regression analyses to examine the associations between social prescribing and several key outcomes. All variables and outcomes were evaluated using a structured, paper-based questionnaire administered face-to-face in the local language by trained interviewers (Supplementary Table S2). The outcomes were assessed using categorical response options. The primary outcomes were overall health status (excellent, good, fair, or poor) and loneliness (often, sometimes, rarely, or never); secondary outcomes were as follows: (I) opportunities for consultation (categories described below); (II) type of consultation partner (categories described below); (III) availability of primary and secondary health-care services (poorly available, moderately available, or highly available); and (IV) unmet needs for health care (yes, or no), long-term care (yes, or no), and social care (yes, or no). Opportunities for consultation were grouped into four domains: (I-1) physical health and functional ability, which included physical problems, pain, mobility and falls, and nutrition; (I-2) mental and cognitive health, which included mental health problems and cognitive decline; (I-3) social and environmental wellbeing, which included financial insecurity and isolation or loneliness; and (I-4) medication and healthcare management which included medication management and access to health care. Types of consultation partners were categorised into two groups: (II-1) health-care professionals, including home doctors and nurses; and (II-2) community supporters, including religious groups, village heads, volunteers, VHSGs, and commune women's councils. All items within these domains were assessed using binary (yes/no) responses. In addition to analysing these domains as aggregated outcomes, supplementary analyses were conducted to examine each specific subcategory within the opportunities for consultation and consultation partner domains.

For each outcome, we estimated odds ratios (ORs) and corresponding 95% confidence intervals (CIs) using logistic regression models. The primary comparison was between Group 1 (recipients of social prescribing) and Group 2 (non-recipients in the same trained districts), with Group 2 serving as the reference. This comparison aimed to explore the associations between receiving social prescribing and key outcomes among recipients. The supplementary analysis compared Group 2 (non-recipients in trained districts) with Group 3 (non-recipients in non-trained districts), with Group 3 serving as the reference. This exploratory analysis aimed to provide initial insights into potential community–level associations related to the presence of trained link workers, even among older adults who did not directly receive social prescribing.

Two models were developed for each comparison: the first model adjusted for age (continuous) and sex (female or male), while the second model included additional adjustments for marital status (single, married, widowed, or divorced), total numbers of household members (continuous), education level (none, pagoda, primary school, secondary school, or higher education), and IDPoor Equity Card holder (yes, or no). P-values were calculated to assess statistical significance. To account for multiple testing, Bonferroni correction was applied for 12 comparisons, yielding an adjusted significance threshold of p < 0.004. Multiple imputation under the assumption of missing at random (MAR) had been planned to address missing data. However, no missing data were ultimately observed in the final dataset, owing to the thorough training provided to interviewers before data collection. All statistical analyses were performed using RStudio 4.4.3.

### Ethics approval

The present study adhered to the principles of the Declaration of Helsinki. Ethical approval for this study has been obtained from the National Ethics Committee for Health Research (NECHR) of the Ministry of Health, Cambodia (No. 215 NECHR). Additional ethical clearance has been granted by the World Health Organization (WHO) Regional Office for the Western Pacific Ethics Review Board (2024.15. KHM.2.AGE). Written informed consent was obtained from all participants, or from legally authorised representatives for participants lacking capacity. Data confidentiality and security were ensured through de-identification, restricted access, and encrypted storage, following established ethical and research governance standards.

### Role of the funding sources

This research is funded by the Healthy Ageing Unit of the WHO Regional Office for the Western Pacific. The funding body provided technical guidance in the development of the monitoring and evaluation protocol and toolkit, and contributed to data analysis and manuscript preparation, in collaboration with the Royal University of Phnom Penh and the WHO Cambodia Country Office.

## Results

### Demographic and contextual characteristics of older adults by social prescribing exposure status

Table 1 summarises the demographic and contextual characteristics of older adults by social prescribing exposure group. The mean age and proportion of female participants were comparable across groups, and most participants were married or widowed. Participants in Group 1 were less likely to live with children (59.2%) or other relatives (40.5%) and had smaller households on average (mean = 4.0, SD = 2.0). Educational attainment was similar across groups, with more than half having completed primary school and about one-third having no formal education. A greater proportion of participants in Group 1 held an IDPoor Equity Card (57.5%) and reported lower household income (<100 USD: 46.2% vs. 42.0% and 36.8% in Groups 2 and 3, respectively). Regarding living conditions, 22.0% of Group 1 households had access only to clean water and 2.0% had both clean water and a sewage system. Access to healthy food, adequate cooking facilities, sufficient living space, environmental protection, and perceived safety were largely comparable across groups.

**Table 1:** Demographic and contextual characteristics of older adults by social prescribing exposure status.

	Group 1		Group 2		Group 3	
Living in the districts with trained link workers	Yes		Yes		No	
Received social prescribing	Yes		No		No	
	**Mean**	**SD**	**Mean**	**SD**	**Mean**	**SD**

Age (mean (SD))	70.3	(6.3)	70.6	(7.0)	70.2	(6.4)
Total number of household (mean (SD))	4.0	(2.0)	4.4	(2.2)	4.7	(2.2)
	**n**	**%**	**n**	**%**	**n**	**%**

Sex (%)						
Female	274	68.5	269	67.2	273	68.2
Male	126	31.5	131	32.8	127	31.8
Marital status (%)						
Single	12	3.0	12	3.0	13	3.2
Married	204	51.0	211	52.8	215	53.8
Widowed	169	42.2	169	42.2	158	39.5
Divorced	15	3.8	8	2.0	14	3.5
Household composition (%)						
Alone	35	8.8	21	5.2	21	5.2
With spouse	204	51.0	211	52.8	215	53.8
With children	237	59.2	267	66.8	267	66.8
With other relatives	162	40.5	215	53.8	232	58.0
Education level (%)						
None	143	35.8	149	37.2	134	33.5
Pagoda	9	2.2	16	4.0	16	4.0
Primary school	198	49.5	190	47.5	208	52.0
Secondary school	41	10.2	37	9.2	36	9.0
Higher education	9	2.2	8	2.0	6	1.5
ID poor card holder (%)	230	57.5	186	46.5	227	56.8
Monthly household income (%)						
Less than 100 USD	185	46.2	168	42.0	147	36.8
100–<300 USD	174	43.5	176	44.0	194	48.5
300–<500 USD	39	9.8	51	12.8	49	12.2
500–<700 USD	2	0.5	2	0.5	7	1.8
700–<900 USD	0	0.0	1	0.2	3	0.8
More than 900 USD	0	0.0	2	0.5	0	0.0
Sewage system at home (%)						
Clean water only	88	22.0	66	16.5	105	26.2
Sewage system only	4	1.0	4	1.0	3	0.8
Both	8	2.0	14	3.5	24	6.0
Neither	300	75.0	316	79.0	268	67.0
Access to healthy food and functional cooking device (%)	261	65.2	276	69.0	268	67.0
Enough space in the house to live comfortably (%)	313	78.2	320	80.0	331	82.8
Home isolated from external stressors (ex. Noise, pollution, hostile weather, storm, flood dry and heat waves) (%)	336	84.0	333	83.2	336	84.0
Safe at home and neighbourhood (%)	384	96.0	382	95.5	389	97.2

Abbreviation: SD, standard deviation; USD, United States Dollars.

### Contents of social prescribing

Among participants in the social prescribing group, 91.0% recalled receiving social prescribing (Table 2). Most heard about it from village health support groups (82.7%) and village heads (76.9%), followed by commune women councils (44.8%) and village members (41.2%). The majority (93.4%) reported a positive first impression. The most common consultation topics were physical problems (83.8%), access to health care (70.9%), and medication management (46.4%). Mental health concerns (28.8%) and financial insecurity (20.1%) were also mentioned, while few consultations addressed nutrition (5.8%) or cognitive decline (7.7%). Social prescribing most often took place at home (55.2%), with some conducted at pagodas or village offices (18.1% each). The most frequently referred services included health facilities (84.3%) and social group/community engagement activities (77.2%). The most frequently referred services included health facilities (84.3%) and social group or community engagement activities (77.2%). Participants were also referred to counselling services at pagodas (20.6%) and mental health and psychosocial support (21.4%). Other less common referrals included educational classes (11.3%), peer support groups (6.9%), and financial advice services (7.4%). Follow-up occurred monthly for 44.0% of participants and quarterly for 31.9%. Reported benefits included health education (69.0%), motivation for health checkups (44.8%), and assistance in times of trouble (35.7%). Comments included gaining the ability to “Understand healthcare” (Participant 01030111), being encouraged to “Go to the doctor on time” (Participant 21041017), and feeling “Warm, helpful, supportive, and healthy” (Participant 17081001). Almost all participants (98.9%) found social prescribing helpful, citing improved understanding of the health system (44.0%) and access to relevant information (47.3%). Participants described receiving “Quick help, encouragement” (Participant 01030505) and practical assistance such as help to “issue them ID poors” (Participant 01030509) and “Help with rice, money, [and] health information” (Participant 01030701). Suggested improvements included better access to healthcare (34.1%), ongoing support over time (33.0%), and financial assistance (28.6%). Participants requested that link workers “Visit more, help more” (Participant 01030506) and asked for material support, with one stating, “Please help by providing food and money so that the elderly can cope” (Participant 01030705). Another suggested a need for more education, asking that officials “Should teach about living and health care more often” (Participant 07040311).

**Table 2:** Contents of social prescribing.

	n	%
Recall of social prescribing (%)	364	91.0
Source of information about social prescribing (%)		
Commune health centre	62	17.0
Commune women council	163	44.8
Village member	150	41.2
Village health support group	301	82.7
Family or direct consultation by older person	7	1.9
Friends	12	3.3
Religious group	38	10.4
Village head	280	76.9
Volunteer	86	23.6
Neighbour	24	6.6
First impression of social prescribing (%)		
Positive	340	93.4
Neutral	24	6.6
Negative	0	0.0
Consultation topic (%)		
Physical problem	305	83.8
Mental problem	105	28.8
Pain	114	31.3
Mobility and falls	51	14.0
Cognitive decline	28	7.7
Medication management	169	46.4
Financial insecurity	73	20.1
Elder abuse	0	0.0
Isolation and loneliness	1	0.3
Access to health care	258	70.9
Nutrition	21	5.8
Location conducted social prescribing (%)		
Home	201	55.2
Pagoda	66	18.1
Health centre	20	5.5
Commune hall	11	3.0
Village office	66	18.1
Referred service (%)		
Health facility	307	84.3
Social group and community engagement	281	77.2
Educational classes	41	11.3
Counselling at pagoda	75	20.6
Mental health support	78	21.4
Peer support	25	6.9
Financial advice	27	7.4
Transportation	5	1.4
Daily life support	62	17.0
Frequency of follow up (%)		
Every month	160	44.0
Every 3 month	116	31.9
Every 6 month	49	13.5
Seldom or never	39	10.7
Benefits of social prescribing (%)		
Health education	251	69.0
Motivation to health checkup	163	44.8
Voluntary community-funded charitable support	72	19.8
Help in time of trouble	130	35.7
Overall helpfulness of social prescribing (%)	360	98.9
Reason (%)		
Understanding the healthcare system	160	44.0
Promoting mutual support	77	21.2
Providing relevant information	172	47.3
Increasing motivation to take action	102	28.0
Free consultation	2	0.5
Suggestion to improve social prescribing (%)		
Improve access to healthcare	124	34.1
Provide financial support	104	28.6
Ensure free access to medicines	31	8.5
Continue support over time	120	33.0
Organise regular meetings	98	26.9

### Findings from the qualitative analysis

Qualitative analysis of the semi-structured interviews identified four principal themes. The first was enhanced health literacy and agency, as participants reported gaining a better understanding of self-care practices such as how to “maintain hygiene, drink boiled water, [and] exercise” (Participant 01030707) and were prompted to “go to the doctor on time” (Participant 21041017). The second theme was practical and navigational support. Link workers provided tangible assistance, such as coordinating hospital visits, helping participants obtain “ID poors” (Participant 01030509), and delivering material aid including “rice, money, [and] health information” (Participant 01030701). A third theme was improved psychosocial well-being. Participants reported positive emotional outcomes, feeling “happier, more supportive, [and] healthier” (Participant 01030506) and “not so lonely” (Participant 22010221), while fostering a sense of mutual support and community. The final theme was a desire for sustained and expanded support. Participants consistently requested more frequent visits and additional financial assistance, with one suggesting a need for “a medical card for the elderly who need to go to the hospital without running out of money” (Participant 17080303), noting that older adults often “can't earn anything” (Participant 01030510).

### Associations between social prescribing exposure and key outcomes in trained districts (Group 1 vs Group 2)

All participants completed the assessments for all key outcomes. Compared to the non-social prescribing group (Group 2), participants in the social prescribing group (Group 1) were more likely to report opportunities to consult about social and environmental well-being (multivariable-adjusted OR (aOR) 1.53, 95% CI 1.02–2.31, *p* = 0.040), although this association did not remain significant after Bonferroni correction (Table 3). No significant differences were observed for other consultation topics, including physical health and functional ability, mental and cognitive health, or medication and healthcare management. In the supplementary analysis (Supplementary Table S3), sub-category–level results indicated that compared to Group 2, participants in Group 1 were more likely to consult about financial insecurity (aOR 1.58, 95% CI 1.05–2.40, *p* = 0.028) and access to health care (aOR 1.50, 95% CI 1.13–1.99, *p* = 0.0055); however, these associations did not withstand Bonferroni correction.

**Table 3:** Associations between opportunities for consultation, availability of health care, unmet needs, health status and exposure of social prescribing: comparison between Group 1 (social prescribing group) and Group 2 (non-social prescribing group, reference) in areas with trained link workers.

	Age and sex adjusted OR (95% CI)	p	Multivariable-adjusted OR^a^ (95% CI)^a^	p
*Opportunity to consult about*				
Physical health and functional ability	0.92 (0.63–1.35)	0.68	0.95 (0.65–1.39)	0.79
Mental and cognitive health	1.06 (0.78–1.43)	0.71	1.02 (0.75–1.38)	0.91
Social and environmental well-being	1.51 (1.01–2.26)	0.044	1.53 (1.02-2.31)	0.040
*Opportunity to consult with*				
Healthcare professionals	1.00 (0.72–1.39)	0.98	1.02 (0.73–1.42)	0.92
Community supporters	1.72 (1.30–2.28)	0.0002	1.65 (1.24–2.20)	0.00054
*Availability of primary and secondary healthcare service*				
Poorly	0.69 (0.51–0.95)	0.021	0.73 (0.53–1.00)	0.048
*Unmet need*				
Healthcare	1.11 (0.81–1.54)	0.51	1.10 (0.79–1.52)	0.58
Long term care	1.14 (0.82–1.59)	0.43	1.15 (0.82–1.60)	0.42
Social welfare need	1.11 (0.82–1.49)	0.51	1.10 (0.81–1.49)	0.53
*Health status*				
Poor overall health status	0.81 (0.59–1.09)	0.17	0.82 (0.60–1.12)	0.22
Feel often lonely	0.64 (0.40–1.02)	0.065	0.60 (0.37–0.97)	0.039

a Adjusted for age, sex, marital status, total number of household members, education level, and ID poor card holder.

In terms of consultation partners, in comparison of Group 2, participants in the social prescribing group (Group 1) were significantly associated with greater opportunities to consult with community supporters (aOR 1.65, 95% CI 1.24–2.20, *p* = 0.00054), which remained statistically significant after Bonferroni correction. In the supplementary analysis, examining individual subcategories of community supporters, social prescribing participation was significantly associated with consultations involving VHSGs (aOR 4.36, 95% CI 3.13–6.15, *p* < 0.0001), volunteers (aOR 3.75, 95% CI 1.95–7.82, *p* = 0.00017), and village heads (aOR 2.16, 95% CI 1.56–3.01, *p* < 0.0001); all three associations remained significant after Bonferroni correction. No significant differences were found for consultations with health-care professionals (aOR 1.02, 95% CI 0.73–1.42, *p* = 0.92). Availability of healthcare was also better among the social prescribing group (Group 1) compared with those without social prescribing (Group 2); Group 1 were less likely to report poor availability of primary and secondary healthcare services (aOR = 0.73, 95% CI: 0.53–1.00, *p* = 0.048), although this association was not statistically significant after Bonferroni correction. No significant differences were observed for unmet needs in healthcare, long-term care, or social welfare. While no significant difference was found in overall health status, participants in the social prescribing group (Group 1) were less likely to report feeling lonely compared to non-social prescribing group (Group 2) (aOR = 0.60, 95% CI: 0.37–0.97, *p* = 0.039), but the association did not remain significant after Bonferroni correction.

### Associations between community link-worker presence and key outcomes among non-recipients (Group 2 vs Group 3)

All participants completed the assessments for all key outcomes. Among participants who had not received social prescribing, those living in areas with trained link workers (Group 2) were more likely to report opportunities to consult with village heads (multivariable-adjusted OR = 1.47, 95% CI: 1.01–2.17, *p* = 0.046) and village health support groups (multivariable-adjusted OR = 1.60, 95% CI: 1.05–2.47, *p* = 0.031), compared to those in areas without trained link workers (Group 3) (Supplementary Table S4). However, these associations were not statistically significant after Bonferroni correction. No significant differences were observed for other factors, such as consultation opportunities for physical or mental health issues, access to healthcare services, unmet needs, or health status.

## Discussion

Social prescribing interventions must be context specific to be effective.^9^ In Cambodia, the National Health Service England's general practitioner led, clinic centred social prescribing model was adapted to fit the local context with few organised community activities and an urgent need for mental health support during the COVID-19 pandemic. Training existing VHSGs as link workers may have the potential to provide an economical, scalable solution that preserved the core principle of connecting people to community resources addressing social determinants of health.

Our findings that suggest this localised, community-based approach was feasible and contextually relevant within the Cambodian setting. The quantitative results showed that participants who received social prescribing were significantly more likely to consult with existing community supporters, including village heads, volunteers and VHSGs. This high level of engagement likely explains the model's resonance and the high satisfaction rate (98.9%) among recipients. By leveraging established and trusted community structures, the intervention was able to provide the timely and hands-on assistance that participants valued, such as being escorted to clinics or helped with IDPoor Equity Card paperwork. This is substantiated by qualitative findings where participants noted that the VHSGs now “take responsibility in the village and encourage, support.”

Furthermore, the intervention may have psychosocial benefits. The quantitative data suggested a potential reduction in reported loneliness among participants in the social prescribing group, but the association did not reach statistical significance after Bonferroni correction. This statistical finding is powerfully supported by our qualitative analysis, where participants reported positive emotional outcomes, feeling “warm and happy” during visits and experiencing a sense of mutual empowerment, speaking of “good health, hope and strength, supporting each other.” This connection between the intervention and reduced loneliness is a critical finding, as the WHO Commission on Social Connection equates weak social ties with major health risk factors such as smoking and obesity.^17^ These results add specific evidence from a low- or middle-income country to the growing body of work showing that enhancing social connection is a vital component of public health.

The study has several limitations. Firstly, its cross-sectional observational design means we can only report on associations and cannot infer causality. The observed relationships may be influenced by confounding factors or selection bias. Furthermore, due to the absence of other social prescribing evaluation studies in Cambodia, we conducted analyses on a large number of outcomes, which raises the possibility of inflated alpha error due to multiple testing. Although Bonferroni correction was applied to control for this, this stringent adjustment may have led to the loss of potentially meaningful associations. Therefore, our findings should be considered exploratory and hypothesis-generating, requiring confirmation in future studies with pre-specified primary outcomes. In addition, some estimates had wide confidence intervals, which may be due to small subgroup sizes and large variability in the data. These results should therefore be interpreted with caution. The findings also rely on self-reported data, which may be affected by recall and social desirability biases, and we lacked objective health utilisation data to corroborate these reports. Finally, while the study included ten provinces, the sample was not nationally representative, which may limit the generalisability of our findings to all older adults in Cambodia.

Nonetheless, the associations with enhanced consultations, perceived access to health and social services, and reduced loneliness are plausible pathways to longer-term health benefits. Future studies should incorporate extended follow-up, objective health metrics, robust cost-effectiveness analyses, and implementation science frameworks that address workforce sustainability and employ digital tools for tracking.

In summary, integrating social prescribing into Cambodia's VHSG network is a feasible and low-cost strategy that operationalises the National Ageing Policy. By linking older adults to local assets it aligns with the first goal of the Policy, and by training younger volunteers it promotes the second goal. With further refinement of the programme and rigorous evaluation, this approach could serve as a scalable template for other low resource contexts confronting the combined pressures of rapid population ageing, accelerating urbanisation, and the rising burden of non communicable diseases.

## Contributors

The authors’ responsibilities were as follows:

Hitomi Kimura: Conceptualisation, writing, and editing.

Sovandara Kao: Study design, data collection, review the manuscript, provide input on mental health and social prescribing.

Sareth Khann: Study design, data collection, review the manuscript, provide input on mental health and social prescribing.

Phan Chan Peou: Review the manuscript, provide input on healthy ageing and mental health in Cambodia.

Daravuth Yel: Review the manuscript, provide input on healthy ageing and mental health in Cambodia.

Ada Moadsiri: Review the manuscript, provide input on healthy ageing and mental health in Cambodia.

Mikiko Kanda: Review the manuscript, provide input on social prescribing and healthy ageing.

Siwon Lee: Conceived the idea, overall supervision, writing, editing, provide input on social prescribing.

Raw data collection and verification were conducted by the Royal University of Phnom Penh (RUPP) research team (Sovandara Kao, Sareth Khann, and Phan Chan Peou), who had full access to the raw data. Hitomi Kimura and Siwon Lee had access to the cleaned and de-identified dataset.

All authors shared responsibility for the decision to submit the manuscript for publication.

## Data sharing statement

Data are available upon request from the authors, subject to approval from the relevant institutional or national ethics board.

## The use of AI and AI-assisted technologies in scientific writing

English language editing support was provided using ChatGPT 5.2 (OpenAI, 2025), an AI-assisted language model, to improve clarity and grammar in the manuscript. The authors reviewed and edited the content to ensure accuracy and appropriateness, and take full responsibility for the final version.

## Declaration of interest

The authors have reported that they have no relationships relevant to the contents of this paper to disclose.
