# Supplementary material for: Bridging health and community: descriptive analysis of social prescribing for older adults in Cambodia
Source: Lancet Reg Health West Pac. 2026 Jan 15;67:101790. doi: 10.1016/j.lanwpc.2025.101790 (PMC12958073; doi:10.1016/j.lanwpc.2025.101790)
Supplement: Supplementary Figures [file mmc1.docx]

Supplementary Figure 1. Sampling framework across trained and non-trained districts


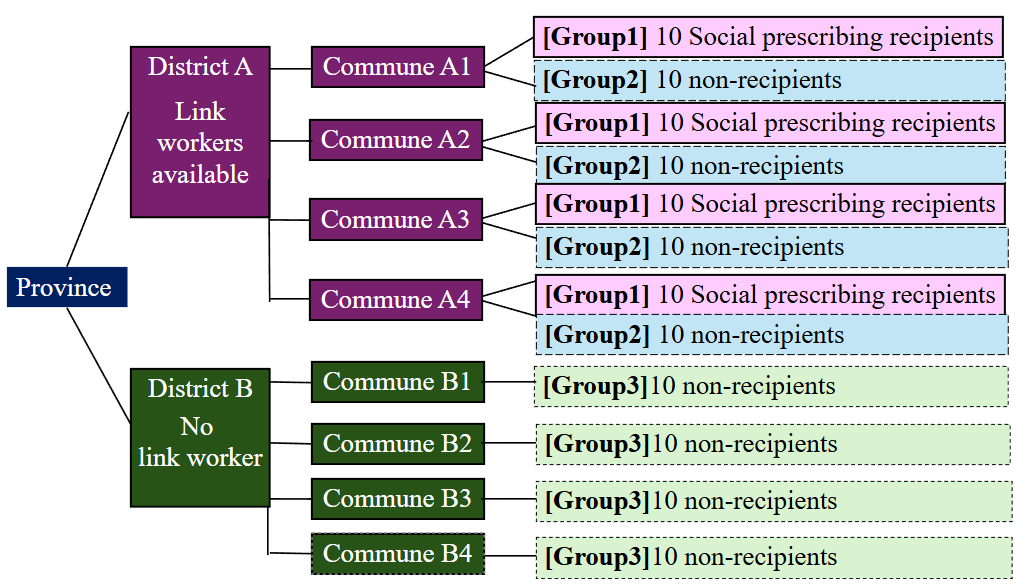


| Supplementary Table 1. Summary of study sites by province and district | | | | | | |
| --- | --- | --- | --- | --- | --- | --- |
| **Province** | **District** | **Group** | **Trained link worker** | **Social prescribing** | **n** |  |
| Banteay Meanchey | Preah Netr Preah | 1 | + | + | 40 |  |
|  |  | 2 | + | - | 40 |  |
|  | Ou Chrov | 3 | - | - | 40 |  |
| Battambang | Sampov Loun | 1 | + | + | 40 |  |
|  |  | 2 | + | - | 40 |  |
|  | Bavel | 3 | - | - | 40 |  |
| Kampong Cham | Prey Chhor | 1 | + | + | 40 |  |
|  |  | 2 | + | - | 40 |  |
|  | Cheung Prey | 3 | - | - | 40 |  |
| Kampot | Angkor Chey | 1 | + | + | 40 |  |
|  |  | 2 | + | - | 40 |  |
|  | Chum Kiri | 3 | - | - | 40 |  |
| Koh Kong | Srae Ambel | 1 | + | + | 40 |  |
|  |  | 2 | + | - | 40 |  |
|  | Kiri Sakor | 3 | - | - | 40 |  |
| Prey Veng | Ba Phnom | 1 | + | + | 40 |  |
|  |  | 2 | + | - | 40 |  |
|  | Kampong Trabaek | 3 | - | - | 40 |  |
| Siem Reap | Chi Kraeng | 1 | + | + | 40 |  |
|  |  | 2 | + | - | 40 |  |
|  | Sot Nikum | 3 | - | - | 40 |  |
| Takeo | Prey Kabbas | 1 | + | + | 40 |  |
|  |  | 2 | + | - | 40 |  |
|  | Angkor Borei | 3 | - | - | 40 |  |
| Kep | Damnak Changáeur | 1 | + | + | 40 |  |
|  |  | 2 | + | - | 40 |  |
|  | Krong Kep | 3 | - | - | 40 |  |
| Tbong Khmum | Memot | 1 | + | + | 40 |  |
|  |  | 2 | + | - | 40 |  |
|  | Ou Reang Ov | 3 | - | - | 40 |  |

Supplementary Table 2. The structured, interviewer-administered questionnaire

| Q1: Do you live in the targeted district?  No: not eligible (If the candidates are not residents of the district, but temporally visitors, they are not eligible.),  Yes: Go to Q2 | - 1. No - 2. Yes |
| --- | --- |
| Q2: Are you aged 60 and over or their family?  No: not eligible  Yes: Go to Q3 | - 1. No - 2. Yes |
| Q3: Are you a resident of the pilot district?  No: Group 3  Yes: Go to Q4 | - 1. No - 2. Yes |
| Q4: Did you get support from trained VHSGs?  No: Group 2  Yes: Group 1 | - 1. No - 2. Yes |
| ・If the respondent is a family member, obtain an answer from the perspective of the older individual in the  family (if the family has multiple older people, the older individual who requires the support most). | |
| ・In this questionnaire, "you" refers to the older person themselves. If the family is answering on their behalf, "you" refers to the older person in the family who needs the most support. | |

Village: .......................... Commune:....................

District:........................... Province:......................

**Participants information**

| 1. ID ....................... | |
| --- | --- |
| 2. The relationship between the respondent and you (the person themselves, spouse, siblings, relatives, others) | 1. the person themselves  2. spouse  3. siblings  4. relatives  5. others............. |

**Demographic Information**

| 1. Age.................... | |
| --- | --- |
| 2. Sex | 1. female  2. male  3. others...... |
| 3. Marital Status: Are you single, married, widowed or divorced? (single, married, widowed, divorced) | 1. single  2. married  3. widowed  4. divorced |
| 4.Household Composition: Do you live alone or with relatives and/or friends? (alone, with spouse, children, other relatives, other) | 1. alone  2. with spouse  3. children  4. other relatives  5. other..... |
| 4-1. How many people in your household? | ............... |
| 5. Number of older people in the family requiring  support for dressing, eating, ambulation, toileting and hygiene? | ............... |
| 6. Who supports you when you have problems? (family, friends, neighbors, colleagues, VHSGs, others) | 1. family  2. friends  3. neighbors  4. colleagues  5. VHSGs  6. others..... |
| 7. Highest level of education: What is your highest level of education (none, pagoda, primary school, secondary school, higher education, other) | 1. none  2. pagoda  3. primary school  4. secondary school  5. higher education  6. other.... |
| 8. What is approximately the total net monthly income from all the sources of income of people living in your household, including yours? | 1. Under $100  2. $100 to < $300  3. $300 to < $500  4. $500 to < $700  5. $700 to < $900  6. $900 and over |
| 9. Do you own an I.D. poor card? | 1. No  2. Yes |
| 10. My home is connected to a satisfactory sewage system or clean water. (1. clean water only 2. sewage system only 3. both 4. neither) | 1. clean water only  2. sewage system only  3. both  4. neither |
| 11. I have access to healthy food and functional  cooking devices. (Yes, No) | 1. No  2. Yes |
| 12. I have enough space in my house to live  comfortably. (Yes, No) | 1. No  2. Yes |
| 13. My house is isolated from external stressors  like noise, pollution, hostile weather, storm, flood, dry and heat waves. (Yes, No) | 1. No  2. Yes |
| 14. I feel safe at home and in my neighborhood.  (Yes, No) | 1. No  2. Yes |

**Contents of social prescribing (skip for non-intervention group)**

| 1. How did you hear about the social prescribing scheme? (from commune health center/ from commune women council/ from village member/ from village health support group/ from family or direct consultation by older people/from friends/ from religious group/ from village heads/ from volunteers/ from neighbors/ others) | 1. commune health center  2. commune women council  3. village member  4. village health support group  5. family or direct consultation by older people  6. friends  7. religious group  8. village heads  9. volunteers  10. neighbors  11. others.............. |
| --- | --- |

| 2. What was your feeling/initial reaction when you learnt about it? (positive/ neutral/ negative) | 1. positive  2. neutral  3. negative |
| --- | --- |
| 3. What did you consult for VHSGs? (physical problem / mental problem /pain/ mobility and falls/ cognitive decline / medication management/ financial insecurity / elder abuse / isolation and loneliness / access to healthcare / nutrition / others) | 1. physical problem  2. mental problem  3. pain  4. mobility and falls  5. cognitive decline  6. medication management  7. financial insecurity  8. isolation and loneliness  9. access to healthcare  10. access to healthcare  11. nutrition  12. others.......... |
| 4. Where did VHSGs conduct social prescribing? (at home / at pagoda / health center / commune hall / village office / others ) | 1. at home  2. at pagoda  3. health center  4. commune hall  5. village office  6. others............. |
| 5. What VHSGs prescribed as social prescribing?(referral to health facility/ social groups and community engagement / educational classes / counseling at pagoda / mental health support / peer support / financial advice / transportation/ daily life support/others) | 1. referral to health facility  2. social groups and community engagement  3. educational classes  4. counseling at pagoda  5. mental health support  6. peer support  7. financial advice  8. transportation  9. daily life support  10. Others…………. |
| 6. How often do you meet VHSGs for follow up? (every month / every 3 month / every 6 month/seldom or never) | 1. every month  2. every 3 month  3. every 6 month  4. seldom or never |

| 7. What benefits did you experience from social prescribing? | ...............................................................  ...............................................................  ...............................................................  ...............................................................  ............................................................... |
| --- | --- |
| 8. Did you find the link worker to be helpful? (Yes, No) | 1. No  2. Yes |
| 8-1. Why/why not? | ...............................................................  ...............................................................  ...............................................................  ...............................................................  ............................................................... |
| 9. How could your experience with social prescribing be improved? | ..............................................................................  ..............................................................................  ..............................................................................  ..............................................................................  .............................................................................. |

**Outcome**

| 1. Unmet health care needs: Do you feel that you need medical care or dental care but can not receive it? (Yes, No) | 1. No  2. Yes |
| --- | --- |
| 1-1. If yes, why? (the care was too expensive, the distance to travel too far, waiting times too long, Others) | 1. the care was too expensive  2. the distance to travel too far  3. waiting times too long  4. Others........... |
| 2. Unmet long-term care needs: Do you need long-term care, such as assistance with daily activities or ongoing health support, but unable to access or receive it? (Yes, No) | 1. No  2. Yes |
| 2-1. If yes, please describe the circumstances. | .............................................................  .............................................................  .............................................................  ............................................................. |

| 3. Unmet social welfare needs: Do you need social welfare needs (such as financial aid, social engagement, housing, or transportation), but  unable to access or receive it? (Yes, No) | 1. No  2. Yes |
| --- | --- |
| 3-1. If yes, please specify which social welfare needs are unmet. (Financial aid, Social engagement, Housing, Transportation, Others) | 1. Financial aid  2. Social engagement  3. Housing  4. Transportation  5. Others............ |
| 4. Are primary and secondary healthcare services and facilities available in your community? (poorly available / moderately available / highly  available) | 1. poorly available  2. moderately available  3. highly available |
| 5. Did you have opportunities to discuss your  health and social care needs in the past year? (Yes / No) | 1. No  2. Yes |
| 5-1. If yes, please specify the needs you  discussed (physical problem / mental problem / pain/ mobility and falls/ cognitive decline / medication management / financial insecurity / elder abuse / isolation and loneliness / access to healthcare / nutrition / others ) | 1. physical problem  2. mental problem  3. pain  4. mobility and falls  5. cognitive decline  6. medication management  7. financial insecurity  8. elder abuse  9. isolation and loneliness  10. access to healthcare  11. nutrition  12. others......... |

| 5-2. If yes, please specify with whom you discussed (with your home doctor / nurses/ with religious group/ with village heads/ with volunteers/ with VHSGs/ with commune women council / others) | 1. with your home doctor  2. nurses  3. with religious group  4. with village heads  5. with volunteers  6. with VHSGs  7. with commune women council  8. others....... |
| --- | --- |
| 6. How would you rate your overall health? (Excellent / Good / Fair / Poor) | 1. Excellent  2. Good  3. Fair  4. Poor |
| 7. Do you feel isolated or lonely? (Often / Sometimes / Rarely / Never) | 1. Often  2. Sometimes  3. Rarely  4. Never |

Supplementary Table 3. Associations between opportunities for consultation (subcategories), availability of health care, unmet needs, health status and exposure of social prescribing:
comparison between Group 1 (social prescribing group) and Group 2 (non-social prescribing group, reference) in areas with trained link workers

|  | Age and sex adjusted OR |  | (95% CI) | | | | p | Multivariable-adjusted OR^a^ | | | (95% CI) | | | | p |
| --- | --- | --- | --- | --- | --- | --- | --- | --- | --- | --- | --- | --- | --- | --- | --- |
| *Opportunity to consult about* |  |  |  |  |  |  |  |  |  |  | |  |  |  |  |
| Physical health and functional ability | 0·92 | ( | 0·63 | - | 1·35 | ) | 0·68 | 0·95 | ( | 0·65 | | - | 1·39 | ) | 0·79 |
| Physical problems | 0·96 | ( | 0·66 | - | 1·39 | ) | 0·82 | 0·97 | ( | 0·67 | | - | 1·41 | ) | 0·87 |
| Pain | 0·85 | ( | 0·64 | - | 1·12 | ) | 0·24 | 0·85 | ( | 0·64 | | - | 1·13 | ) | 0·28 |
| Mobility and fall | 0·95 | ( | 0·65 | - | 1·40 | ) | 0·81 | 0·94 | ( | 0·64 | | - | 1·38 | ) | 0·74 |
| Nutrition | 1·90 | ( | 0·97 | - | 3·90 | ) | 0·068 | 1·84 | ( | 0·93 | | - | 3·80 | ) | 0·087 |
| Mental and cognitive health | 1·06 | ( | 0·78 | - | 1·43 | ) | 0·71 | 1·02 | ( | 0·75 | | - | 1·38 | ) | 0·91 |
| Mental problems | 1·20 | ( | 0·85 | - | 1·71 | ) | 0·30 | 1·16 | ( | 0·81 | | - | 1·66 | ) | 0·42 |
| Cognitive decline | 1·14 | ( | 0·58 | - | 2·24 | ) | 0·71 | 1·21 | ( | 0·61 | | - | 2·40 | ) | 0·59 |
| Social and environmental well-being | 1·51 | ( | 1·01 | - | 2·26 | ) | 0·044 | 1·53 | ( | 1·02 | | - | 2·31 | ) | 0·040 |
| Financial insecurity | 1·55 | ( | 1·04 | - | 2·34 | ) | 0·033 | 1·58 | ( | 1·05 | | - | 2·40 | ) | 0·028 |
| Isolation and loneliness | 0·33 | ( | 0·02 | - | 2·61 | ) | 0·34 | 0·30 | ( | 0·01 | | - | 2·91 | ) | 0·33 |
| Medication and healthcare management | 1·17 | ( | 0·80 | - | 1·70 | ) | 0·41 | 1·18 | ( | 0·81 | | - | 1·72 | ) | 0·40 |
| Medication management | 0·95 | ( | 0·70 | - | 1·30 | ) | 0·76 | 0·98 | ( | 0·72 | | - | 1·35 | ) | 0·92 |
| Access to health care | 1·56 | ( | 1·18 | - | 2·07 | ) | 0·0019 | 1·50 | ( | 1·13 | | - | 1·99 | ) | 0·0055 |
| *Opportunity to consult with* |  |  |  |  |  |  |  |  |  |  | |  |  |  |  |
| Healthcare professionals | 1·00 | ( | 0·72 | - | 1·39 | ) | 0·98 | 1·02 | ( | 0·73 | | - | 1·42 | ) | 0·92 |
| Home doctor | 1·09 | ( | 0·76 | - | 1·56 | ) | 0·65 | 1·11 | ( | 0·77 | | - | 1·59 | ) | 0·58 |
| Nurse | 0·93 | ( | 0·69 | - | 1·26 | ) | 0·65 | 0·95 | ( | 0·70 | | - | 1·29 | ) | 0·73 |
| Community supporters | 1·72 | ( | 1·30 | - | 2·28 | ) | 0·0002 | 1·65 | ( | 1·24 | | - | 2·20 | ) | 0·00054 |
| Religious group | 1·19 | ( | 0·89 | - | 1·60 | ) | 0·24 | 1·17 | ( | 0·87 | | - | 1·58 | ) | 0·29 |
| Village head | 2·17 | ( | 1·57 | - | 3·01 | ) | <0·0001 | 2·16 | ( | 1·56 | | - | 3·01 | ) | <0·0001 |
| Volunteer | 3·94 | ( | 2·06 | - | 8·18 | ) | 0·0001 | 3·75 | ( | 1·95 | | - | 7·82 | ) | 0·00017 |
| Village health support group | 4·59 | ( | 3·30 | - | 6·46 | ) | <0·0001 | 4·36 | ( | 3·13 | | - | 6·15 | ) | <0·0001 |
| Commune women council | 1·53 | ( | 0·77 | - | 3·12 | ) | 0·23 | 1·44 | ( | 0·72 | | - | 2·95 | ) | 0·31 |
| *Availability of primary and secondary healthcare service* |  |  |  |  |  |  |  |  |  |  | |  |  |  |  |
| Poorly | 0·69 | ( | 0·51 | - | 0·95 | ) | 0·021 | 0·73 | ( | 0·53 | | - | 1·00 | ) | 0·048 |
| *Unmet need* |  |  |  |  |  |  |  |  |  |  | |  |  |  |  |
| Healthcare | 1·11 | ( | 0·81 | - | 1·54 | ) | 0·51 | 1·10 | ( | 0·79 | | - | 1·52 | ) | 0·58 |
| Long term care | 1·14 | ( | 0·82 | - | 1·59 | ) | 0·43 | 1·15 | ( | 0·82 | | - | 1·60 | ) | 0·42 |
| Social welfare need | 1·11 | ( | 0·82 | - | 1·49 | ) | 0·51 | 1·10 | ( | 0·81 | | - | 1·49 | ) | 0·53 |
| *Health status* |  |  |  |  |  |  |  |  |  |  | |  |  |  |  |
| Poor overall health status | 0·81 | ( | 0·59 | - | 1·09 | ) | 0·17 | 0·82 | ( | 0·60 | | - | 1·12 | ) | 0·22 |
| Feel often lonely | 0·64 | ( | 0·40 | - | 1·02 | ) | 0·065 | 0·60 | ( | 0·37 | | - | 0·97 | ) | 0·039 |

a Adjusted for age, sex, marital status, total number of household members, education level, and ID poor card holder

Supplementary Table 4. Associations between opportunities for consultation, availability of health care, unmet needs, health status and presence of link workers in the community~~:~~
comparison between Group 2 (residents with trained link workers) and Group 3 (residents without trained link workers, reference) among non-social prescribing group

|  | Age and sex adjusted OR |  | (95% CI) | | |  | p | Multivariable-adjusted OR^a^ |  | (95% CI) | | |  | p |  |
| --- | --- | --- | --- | --- | --- | --- | --- | --- | --- | --- | --- | --- | --- | --- | --- |
| *Opportunity to consult about* |  |  |  |  |  |  |  |  |  |  |  |  |  |  |  |
| Physical health and functional ability | 1·04 | ( | 0·71 | - | 1·52 | ) | 0·85 | 1·01 | ( | 0·69 | - | 1·48 | ) | 0·97 |  |
| Physical problems | 1·06 | ( | 0·73 | - | 1·52 | ) | 0·77 | 1·02 | ( | 0·71 | - | 1·48 | ) | 0·90 |  |
| Pain | 1·06 | ( | 0·80 | - | 1·40 | ) | 0·68 | 1·06 | ( | 0·80 | - | 1·40 | ) | 0·70 |  |
| Mobility and fall | 1·04 | ( | 0·71 | - | 1·53 | ) | 0·82 | 1·06 | ( | 0·72 | - | 1·56 | ) | 0·77 |  |
| Nutrition | 0·64 | ( | 0·31 | - | 1·29 | ) | 0·22 | 0·67 | ( | 0·32 | - | 1·35 | ) | 0·27 |  |
| Mental and cognitive health | 1·10 | ( | 0·81 | - | 1·49 | ) | 0·55 | 1·09 | ( | 0·80 | - | 1·49 | ) | 0·58 |  |
| Mental problems | 1·42 | ( | 0·97 | - | 2·10 | ) | 0·071 | 1·47 | ( | 1·00 | - | 2·17 | ) | 0·053 |  |
| Cognitive decline | 0·82 | ( | 0·42 | - | 1·60 | ) | 0·57 | 0·83 | ( | 0·42 | - | 1·63 | ) | 0·59 |  |
| Social and environmental well-being | 0·98 | ( | 0.64 | - | 1.49 | ) | 0·91 | 0·96 | ( | 0·62 | - | 1·48 | ) | 0·85 |  |
| Financial insecurity | 0·98 | ( | 0·63 | - | 1·51 | ) | 0·92 | 0·96 | ( | 0·62 | - | 1·49 | ) | 0·87 |  |
| Isolation and loneliness | 0·74 | ( | 0·14 | - | 3·39 | ) | 0·70 | 0·70 | ( | 0·13 | - | 3·38 | ) | 0·66 |  |
| Social and environmental well-being | 1·05 | ( | 0·74 | - | 1·51 | ) | 0·77 | 1·08 | ( | 0·75 | - | 1·56 | ) | 0·68 |  |
| Medication management | 0·95 | ( | 0·70 | - | 1·31 | ) | 0·77 | 0·94 | ( | 0·69 | - | 1·29 | ) | 0·71 |  |
| Access to health care | 0·91 | ( | 0·69 | - | 1·20 | ) | 0·51 | 0·95 | ( | 0·71 | - | 1·26 | ) | 0·71 |  |
| *Opportunity to consult with* |  |  |  |  |  |  |  |  |  |  |  |  |  |  |  |
| Healthcare professionals | 1·10 | ( | 0·80 | - | 1·53 | ) | 0·55 | 1·09 | ( | 0·79 | - | 1·51 | ) | 0·60 |  |
| Home doctor | 0·92 | ( | 0·64 | - | 1·32 | ) | 0·66 | 0·94 | ( | 0·65 | - | 1·35 | ) | 0·73 |  |
| Nurse | 1·34 | ( | 1·00 | - | 1·81 | ) | 0·054 | 1·33 | ( | 0·99 | - | 1·81 | ) | 0·061 |  |
| Community supporters | 0·98 | ( | 0·75 | - | 1·30 | ) | 0·91 | 1.00 | ( | 0·75 | - | 1·32 | ) | 0·99 |  |
| Religious group | 0·78 | ( | 0·58 | - | 1·04 | ) | 0·088 | 0·78 | ( | 0·58 | - | 1·04 | ) | 0·092 |  |
| Village head | 1·46 | ( | 1·01 | - | 2·14 | ) | 0·048 | 1·47 | ( | 1·01 | - | 2·17 | ) | 0·046 |  |
| Volunteer | 1·39 | ( | 0·55 | - | 3·62 | ) | 0·49 | 1·29 | ( | 0·51 | - | 3·40 | ) | 0·60 |  |
| Village health support group | 1·58 | ( | 1·04 | - | 2·42 | ) | 0·033 | 1·60 | ( | 1·05 | - | 2·47 | ) | 0·031 |  |
| Commune women council | 1·44 | ( | 0·64 | - | 3·39 | ) | 0·38 | 1·64 | ( | 0·72 | - | 3·90 | ) | 0·25 |  |
| *Availability of primary and secondary healthcare service* |  |  |  |  |  |  |  |  |  |  |  |  |  |  |  |
| Poorly | 1·08 | ( | 0·80 | - | 1·47 | ) | 0·60 | 1·07 | ( | 0·79 | - | 1·45 | ) | 0·67 |  |
| *Unmet need* |  |  |  |  |  |  |  |  |  |  |  |  |  |  |  |
| Healthcare | 0·95 | ( | 0·69 | - | 1·32 | ) | 0·76 | 0·98 | ( | 0·71 | - | 1·37 | ) | 0·93 |  |
| Long term care | 1·18 | ( | 0·84 | - | 1·67 | ) | 0·34 | 1·19 | ( | 0·84 | - | 1·69 | ) | 0·32 |  |
| Social welfare need | 1·18 | ( | 0·88 | - | 1·58 | ) | 0·27 | 1·19 | ( | 0·89 | - | 1·61 | ) | 0·25 |  |
| *Health status* |  |  |  |  |  |  |  |  |  |  |  |  |  |  |  |
| Poor overall health status | 1·18 | ( | 0·87 | - | 1·59 | ) | 0·28 | 1·17 | ( | 0·86 | - | 1·58 | ) | 0·32 |  |
| Feel often lonely | 1·01 | ( | 0·66 | - | 1·55 | ) | 0·96 | 1·01 | ( | 0·65 | - | 1·57 | ) | 0·96 |  |

a Adjusted for age, sex, marital status, total number of household members, education level, and ID poor card holder
